# Emergence of the coexistence of *optrA* and *fexA* in *Campylobacter coli* strains isolated from the market

**DOI:** 10.1128/spectrum.02941-24

**Published:** 2025-09-25

**Authors:** Xiaoli Wang, Xue Zheng, Jiangang Ma, Xiujuan Wang, Guoping Zhao, Biao Tang

**Affiliations:** 1 Key Laboratory of Systems Health Science of Zhejiang Province, School of Life Science, Hangzhou Institute for Advanced Study, University of Chinese Academy of Sciences 638898, Hangzhou, China; 2 State Key Laboratory for Managing Biotic and Chemical Threats to the Quality and Safety of Agro-Products & Institute of Agro-product Safety and Nutrition, Zhejiang Academy of Agricultural Sciences 74561, Hangzhou, Zhejiang, China; 3 Xianghu Laboratory 665999 , Hangzhou, China; 4 China MOA Laboratory for Risk Assessment of Quality and Safety of Livestock and Poultry Products, Huazhong Agricultural University 47895, Wuhan, China; University of Saskatchewan, Saskatoon, Saskatchewan, Canada

**Keywords:** *Campylobacter coli*, antimicrobial resistance, *optrA*, *fexA*

## LETTER

As an important foodborne bacterium, *Campylobacter* encounters diverse antimicrobials during livestock production. Consequently, it has evolved different resistance strategies, notably the acquisition of multidrug-resistance genomic islands , to enhance survival under antimicrobial pressure (1, 2). The *fexA* gene mediates resistance to florfenicol (2–4), whereas *optrA* not only confers phenicol resistance but also elevates resistance to the oxazolidinone drug linezolid (3, 5, 6). Accordingly, *Campylobacter* strains harboring both *fexA* and *optrA* raise important public health concerns due to their expanded resistance profile (7). In this study, the co-occurrence of *optrA* and *fexA* was identified in *Campylobacter coli* isolates obtained from retail meat. Complete genome sequencing was performed to characterize the genetic context of these resistance genes.

A total of 88 *Campylobacter* strains were isolated from 417 samples collected in 2021 across four cities in Zhejiang Province, China (Fig. S1). Among these, 37 were identified as *Campylobacter jejuni* and 51 as *C. coli* isolates (Table S1). The isolates exhibited high resistance rates to nalidixic acid (95.45%), ciprofloxacin (93.18%), and tetracycline (97.73%), compared to other tested antibiotics. The lowest resistance rates were observed for erythromycin and telithromycin, both at 39.77% (Fig. S2A). In total, 25 distinct antimicrobial resistance (AMR) patterns were identified among the isolates (Table S2). The predominant AMR pattern of *Campylobacter* isolates was ciprofloxacin (CIP)-nalidixic acid (NAL)-gentamicin (GEN)-tetracycline (TET)-clindamycin (CLI)-erythromycin (ERY)-azithromycin (AZM)-telithromycin (TEL)-florfenicol (FFC). Fig. S2B illustrates the number and proportion of isolates resistant to different classes of antibiotics. Specifically, 3.41% (3/88) of the isolates were susceptible to all tested antibiotics, 1.13% (1/88) were resistant to a single class, and 75.00% (66/88) exhibited multidrug resistance, defined as resistance to three or more antibiotic classes.

**Fig 1:**
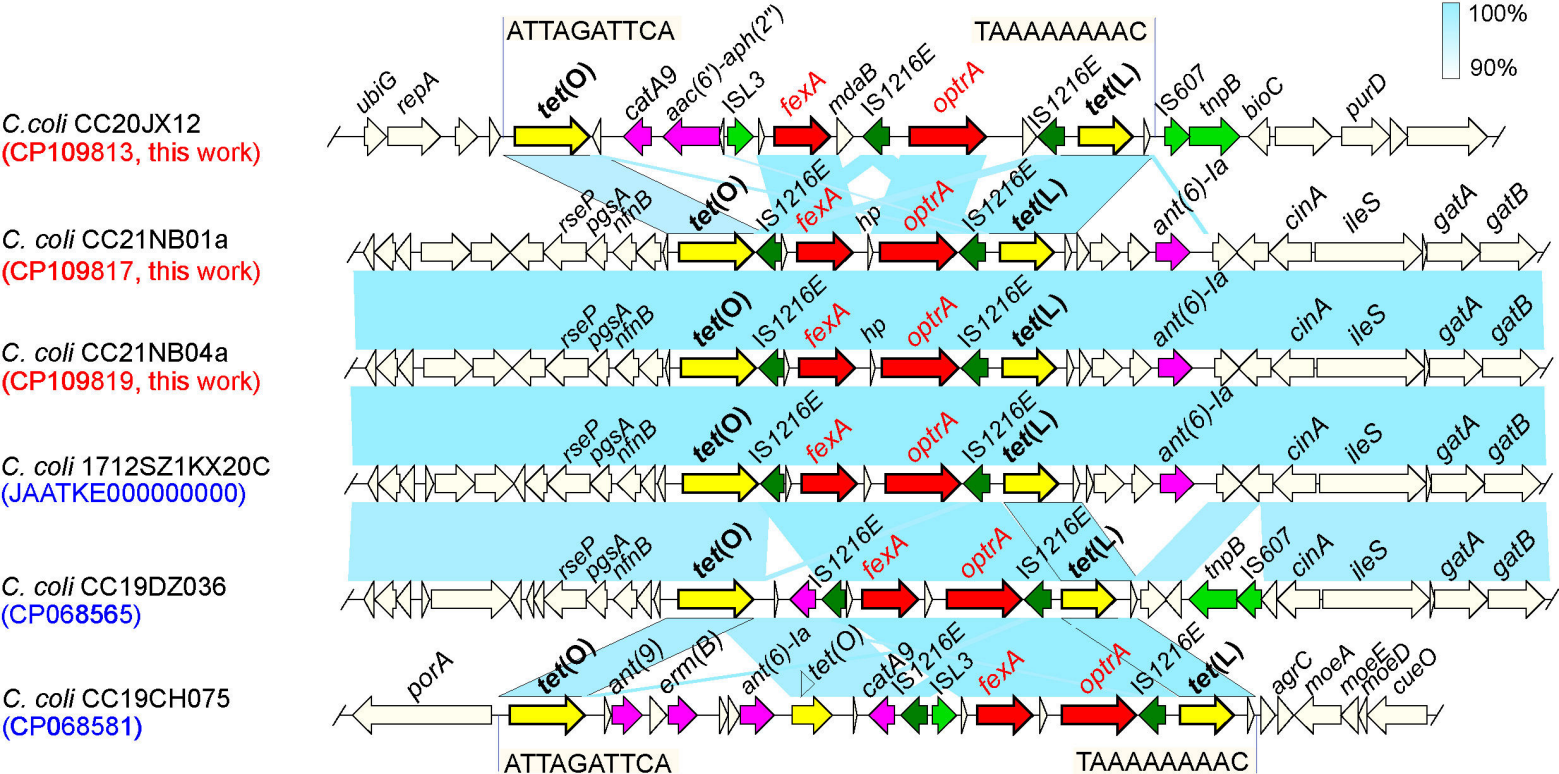
Genetic environment of *optrA* and *fexA* genes in *C. coli* genomes. Arrows indicate the positions of the genes and the direction. Regions with >90% homology are marked with blue shading. Genes are distinguished by different colors.

The *fexA* and *optrA* genes were detected by polymerase chain reaction (PCR) amplification (Table S3 and Fig. S3). Among the *fexA*-positive strains, 3 (9.38%; 3/32) were *C. jejuni*, while 29 (90.62%; 29/32) were *C. coli*. Notably, all three isolates co-harboring *optrA* and *fexA* were *C. coli*. For detailed characterization of these isolates and their resistance gene environments, we generated complete genome assemblies using a hybrid strategy that integrated Illumina short reads with Oxford Nanopore long reads. (2, 8) (Table 1). The genetic environment of *optrA* and *fexA* genes in *C. coli* genomes appeared to be conserved (Fig. 1). Interestingly, the *mdaB*–IS*1216E* structure observed in strain CC20J × 12 has not been reported previously. *In silico* MLST analysis showed that seven *optrA–fexA*–positive isolates (three from our collection and four from GenBank) were assigned to three sequence types: ST825, ST828, and a novel sequence type (designated ST112510 in this study) (Fig. S4).

**TABLE 1:** The AMR and genome characteristics of three optrA+ *fexA*+ isolates in this study^*a*^

Antibiotics	Strains		
	CJ21NB01a	CC21NB04a	CC20J × 12
CIP	64	64	32
NAL	128	>128	>128
GEN	64	64	>64
TET	>64	>64	>64
CLI	8	8	>32
ERY	>64	>64	64
AZM	>32	>32	>32
TEL	32	32	32
florfenFFC	32	32	>32
acquired AMR genes	*ant (6)-la*, *aac(3')-III*, *aac(6')-aph(2"*), *optrA*, *fexA*, *tet*(O), *tet*(L)	*ant (6)-la*, *aac(3')-III*, *aac(6')-aph(2"*), *optrA*, *fexA*, *tet*(O), *tet*(L)	*ant (6)-la*, *aac(3')-III*, aac(6')-aph(2"), optrA, *fexA*, *tet*(O), *tet*(L), *bla*_OXA-61_, *erm*(B),*catA9*
mutation	*gyrA*_2 (p.T86I)	*gyrA*_2 (p.T86I)	*gyrA*_2 (p.T86I)
	23S (2075A > G)	23S (2075A > G)	
sequence type	ST828	ST828	ST112510
GC content	31.45%	31.45%	31.47%
genome size (bp)	chr: 1,778,927 pCC21NB01-51K: 51,931	chr: 1,779,054 pCC21NB04-51K: 51,931	chr: 1,698,077 pCC20J×12–60K: 60,040 pCC20J×12–27K: 27,725 pCC20J×12–5K: 5,363

^*a*^ Gentamicin (GEN), nalidixic acid (NAL), ciprofloxacin (CIP), erythromycin (ERY), azithromycin (AZI), tetracycline (TET), florfenicol (FFC), telithromycin (TEL) and clindamycin (CLI).

The *fexA-hp-optrA* sequence was found to be highly conserved based on BLAST analysis against the GenBank database (Fig. S5). A total of 127 *optrA-hp-fexA* sequences (approximately 4.3 kb) were extracted from our *Campylobacter* isolates and publicly available genomes in GenBank. Multiple sequence alignment was performed, and a maximum likelihood phylogenetic tree was constructed to assess their evolutionary relationship. This genetic fragment was identified in *Enterococcus faecalis*, *Enterococcus faecium*, *Staphylococcus* spp., and *Vagococcus lutrae*, with *E. faecalis* being the predominant host. Notably, the sequence was detected in *E. faecalis* strains C25, AR_0780, and CVM_N48037F, each harboring two copies of the element. (Fig. S5). Phylogenetic analysis revealed that eight *Campylobacter* strains clustered within the same branch. Interestingly, *C. jejuni* strain ZS007 grouped closely with *Enterococcus* species, showing no sequence divergence and was clearly distinct from other *Campylobacter* isolates (Fig. S5). These findings provide molecular evidence supporting the horizontal transfer of this resistance gene fragment from *Enterococcus* to *Campylobacter*.

*Campylobacter* ranks among the most important foodborne pathogens, and its rising AMR represents an increasing public health challenge (9, 10). In this study, we report the detection of coexisting resistance genes *optrA* and *fexA* in *C. coli* strains isolated from retail meat. Although this gene arrangement has been identified previously, its presence in *Campylobacter* from meat sources remains relatively uncommon and thus noteworthy. Phylogenetic analysis of 127 conserved sequences within the *optrA-hp-fexA* region suggested that this gene arrangement may have originated from *Enterococcus* spp. Collectively, our findings underscore the urgent need to monitor and control the spread of key AMR genes within the food chain to mitigate potential risks to human health.

## Data Availability

The whole genome sequences of *C. coli* strains CC20JX12, CC21NB01a, and CC21NB04a have been deposited in the GenBank database under accession numbers CP109813-CP109816, CP109817-CP109818, and CP109819-CP109820, respectively.

## SUPPLEMENTAL MATERIAL

Supplemental material spectrum.02941-24-s0001.docx Figure S1 to S5; Tables S1 to S3.

ASM does not own the copyrights to Supplemental Material that may be linked to, or accessed through, an article. The authors have granted ASM a non-exclusive, world-wide license to publish the Supplemental Material files. Please contact the corresponding author directly for reuse.
