# Supplementary material for: Emergence of the coexistence of optrA and fexA in Campylobacter coli strains isolated from the market
Source: Microbiol Spectr. 2025 Sep 25;13(11):e02941-24. doi: 10.1128/spectrum.02941-24 (PMC12584668; doi:10.1128/spectrum.02941-24)
Supplement: Supplemental material — Figure S1 to S5; Tables S1 to S3. [file spectrum.02941-24-s0001.docx]

**
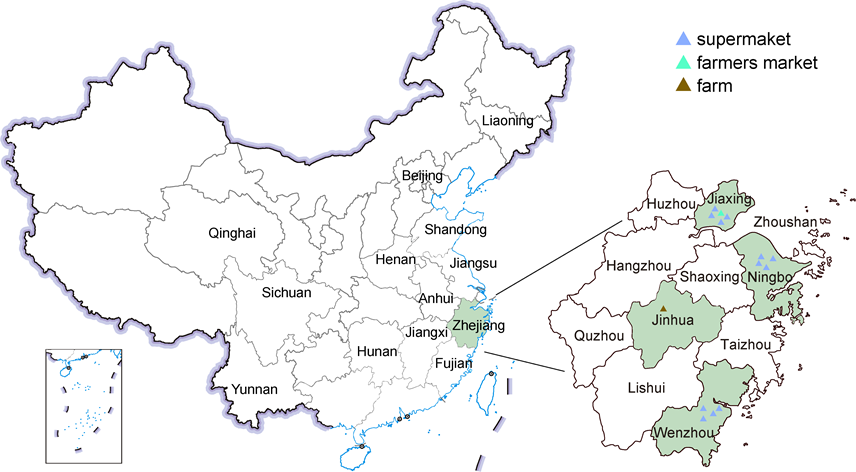
Figure S1**. Map of sampling areas in Zhejiang provinces, China. The sampling sources are denoted with triangles.


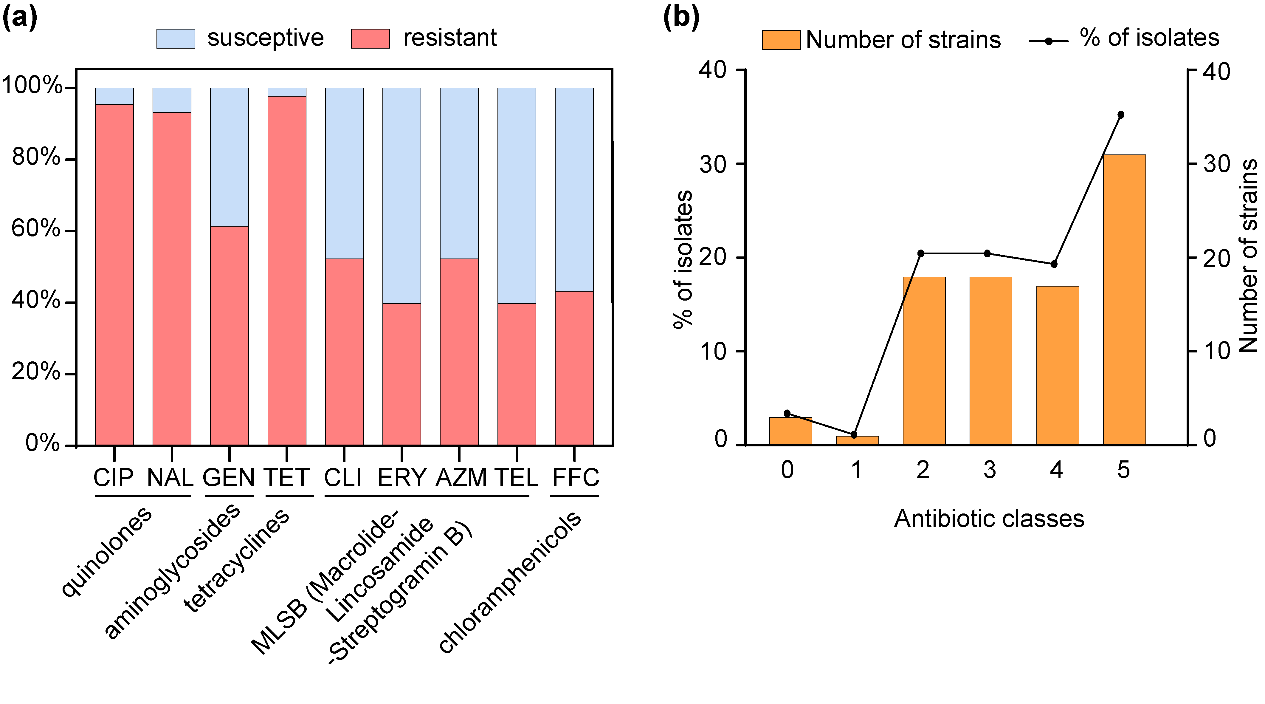


**Figure S2**. AMR rates of *Campylobacter* isolates in this study. (A) The prevalence of antimicrobial-resistant isolates for each individual antibiotic. (B) The distribution of *Campylobacter* isolates with different AMR phenotypes.


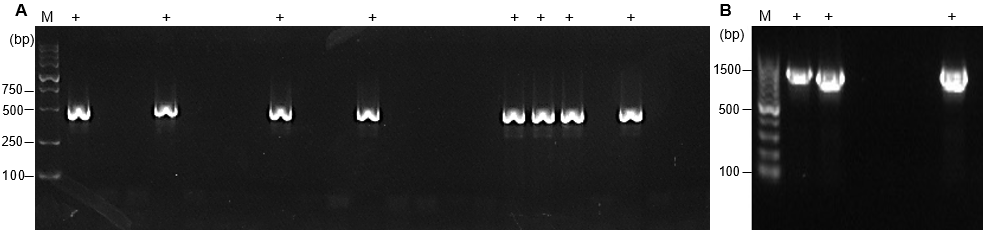


**Figure S3**. PCR amplification of *fexA* and *optrA* genes. (**A**) Detection of *fexA* by PCR. Amplification of the *fexA* gene using the forward primer TTTTAATGATGGTACTCTCCCT and reverse primer GGTAACGCGTAGTAGGCACCAA, yielding the expected 529-bp fragment. (**B**) Detection of *optrA* by PCR. Amplification of the *optrA* gene using the forward primer AGGTGGTCAGCGAACTAA and reverse primer ATCAACTGTTCCCATTCA, yielding the expected 1,395-bp fragment. “M” denotes the DNA marker; lanes labeled with “+” indicate positive results.


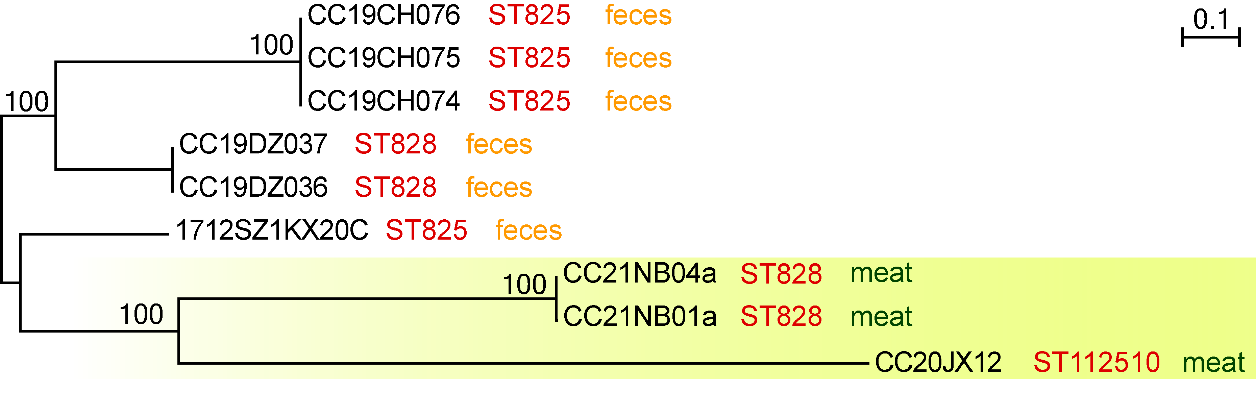


**Figure S4**. SNP tree of *C. coli* strains co-carrying *fexA* and *optrA* genes based on whole genome sequences. The complete genome sequences of strains CC21NB01a, CC21NB04a, and CC20JX12 were sequenced in this study.


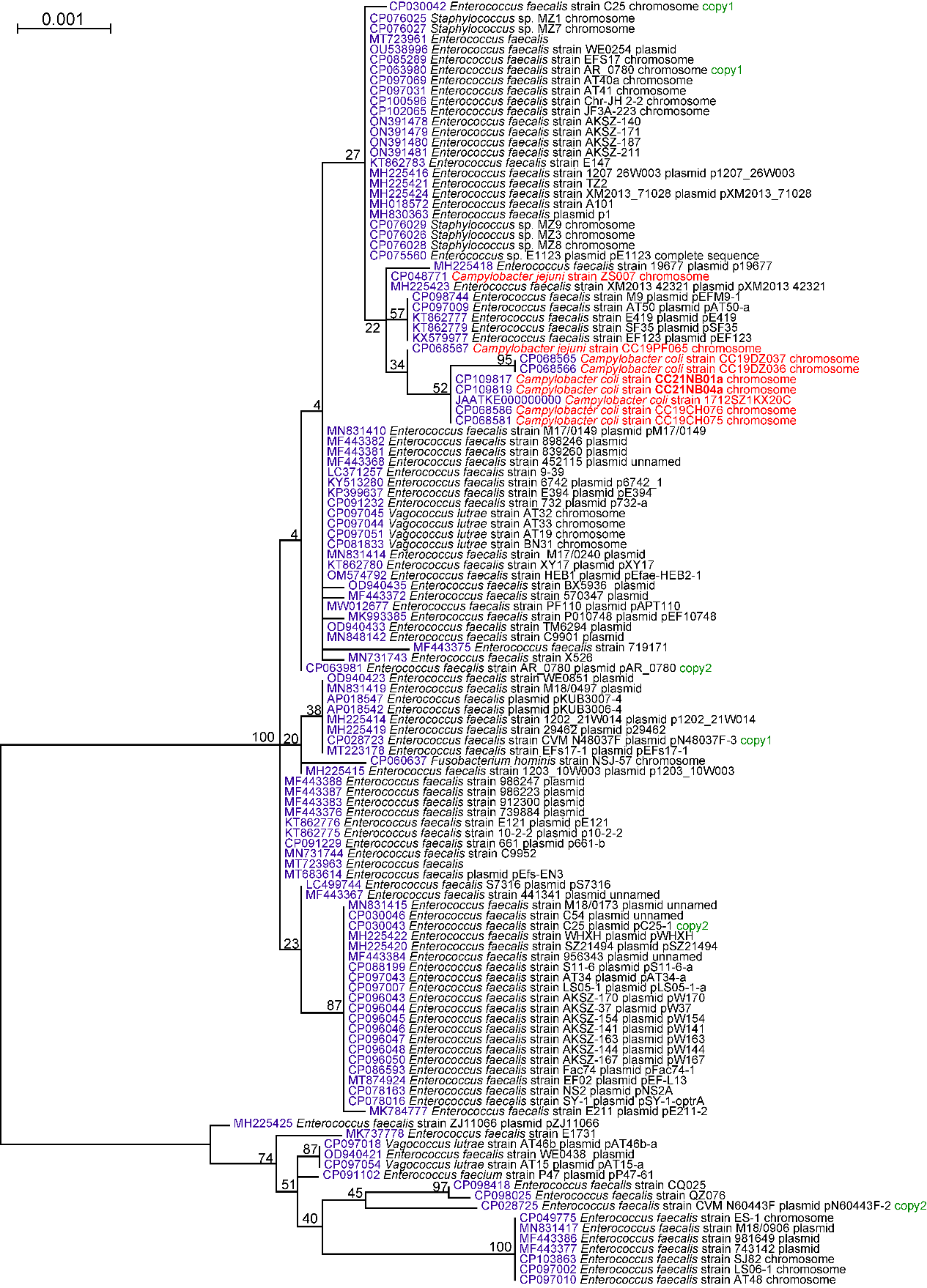


**Figure S5**. Phylogeny of the *fexA*-*hp*-*optrA* sequence. The analysis involved 127 nucleotide sequences. Maximum likelihood method was used to infer the phylogeny of *fexA*-*hp*-*optrA* segments. This phylogenetic tree relied on 1000 re-sampled datasets. *Campylobacter* strains were marked red. The strains with two copies of *fexA*-*hp*-*optrA* are marked green.

**Table S1.** The prevalence of *Campylobacter* strains isolated from food animal chain in this study

| Source | | | No. of samples | No. of *Campylobacter* strains  (isolation rate %) | | |
| --- | --- | --- | --- | --- | --- | --- |
|  |  |  |  | *C. jejuni* | *C. coli* | Total |
| supermarket | Jiaxing | meat | 95 | 8 (8.42%) | 2 (2.11%) | 10 (10.53%) |
|  | Ningbo | meat | 95 | 4 (4.21%) | 3 (3.16%) | 7 (7.37%) |
|  | Wenzhou | meat | 86 | 0 (0.00) | 6 (6.98%) | 6 (6.98%) |
| farmers market | Jiaxing | meat | 70 | 23 (32.86%) | 36 (51.43%) | 59 (84.29%) |
|  | Ningbo | meat | 10 | 0 (0.00) | 4 (40.00%) | 4 (40.00%) |
| farm | Jinhua | swab | 61 | 2 (3.28%) | 0 (0.00) | 2 (3.28%) |
| **Total** |  |  | **417** | **37 (8.87%)** | **51 (12.23%)** | **88 (21.10%)** |

**Table S2.** The resistance patterns of 88 *Campylobacter* strains

| **Antimicrobials number** | **antibiotic resistance pattern** | **isolates** | **Percentage (%)** |
| --- | --- | --- | --- |
| 9 | CIP-NAL-GEN-TET-CLI-ERY-AZM-TEL-FFC | 23 | 26.14% |
| 3 | CIP-NAL-TET | 15 | 17.05% |
| 8 | CIP-NAL-GEN-TET-CLI-ERY-AZM-TEL | 7 | 7.95% |
| 4 | CIP-NAL-TET-FFC | 5 | 5.68% |
| 4 | CIP-NAL-GEN-CIP | 5 | 5.68% |
| 7 | CIP-NAL-GEN-TET-CLI-AZM-FFC | 5 | 5.68% |
| 2 | CIP-NAL | 3 | 3.41% |
| 0 | --------- | 3 | 3.41% |
| 6 | CIP-NAL-GEN-TET-CLI-AZM | 2 | 2.27% |
| 5 | CIP-NAL-GEN-TET-AZM | 2 | 2.27% |
| 5 | CIP-NAL-GEN-TET-CLI | 2 | 2.27% |
| 5 | CIP-NAL-GEN-TET-FFC | 2 | 2.27% |
| 1 | CIP | 1 | 1.14% |
| 2 | CIP-TET | 1 | 1.14% |
| 2 | NAL-TET | 1 | 1.14% |
| 3 | CIP-NAL-FFC | 1 | 1.14% |
| 4 | CIP-NAL-TET-TEL | 1 | 1.14% |
| 4 | CIP-NAL-TET-AZM | 1 | 1.14% |
| 4 | CIP-NAL-GEN-CLI | 1 | 1.14% |
| 5 | CIP-NAL-GEN-TET-TEL | 1 | 1.14% |
| 5 | CIP-NAL-GEN-CLI-AZM | 1 | 1.14% |
| 5 | CIP-NAL-TET-CLI-AZM | 1 | 1.14% |
| 7 | CIP-NAL-TET-CLI-ERY-AZM-TEL | 1 | 1.14% |
| 7 | CIP-NAL-GEN-TET-CLI-ERY-AZM | 1 | 1.14% |
| 8 | CIP-GEN-TET-CLI-ERY-AZM-TEL-FFC- | 1 | 1.14% |
| 8 | CIP-NAL-GEN-TET-CLI-AZM-TEL-FFC | 1 | 1.14% |

**Table S3.** The primers used for PCR in this study

| gene | sequence | the length of PCR products (bp) |
| --- | --- | --- |
| *optrA* | F: AGGTGGTCAGCGAACTAA | 1,395 |
|  | R: ATCAACTGTTCCCATTCA |  |
| *fexA* | F: TTTTAATGATGGTACTCTCCCT | 529 |
|  | R: GGTAACGCGTAGTAGGCACCAA |  |
